# Analysis of the Expression of Repetitive DNA Elements in Osteosarcoma

**DOI:** 10.3389/fgene.2017.00193

**Published:** 2017-11-30

**Authors:** Xuan D. Ho, Hoang G. Nguyen, Le H. Trinh, Ene Reimann, Ele Prans, Gea Kõks, Katre Maasalu, Van Q. Le, Van H. Nguyen, Nghi T. N. Le, Phuong Phung, Aare Märtson, Freddy Lattekivi, Sulev Kõks

**Affiliations:** ^1^Department of Oncology, College of Medicine and Pharmacy, Hue University, Hue, Vietnam; ^2^Department of Pathophysiology, University of Tartu, Tartu, Estonia; ^3^Department of Oncology, Hanoi Medical University, Hanoi, Vietnam; ^4^Department of Reproductive Biology, Estonian University of Life Sciences, Tartu, Estonia; ^5^Department of Traumatology and Orthopedics, University of Tartu, Tartu, Estonia; ^6^Clinic of Traumatology and Orthopaedics of Tartu University Hospital, Tartu, Estonia; ^7^Department of Orthopedics, College of Medicine and Pharmacy, Hue University, Hue, Vietnam

**Keywords:** osteosarcoma, human endogenous retrovirus, sequence, satellite, repetitive elements, transposable elements

## Abstract

Osteosarcoma (OS) is a rare malignant bone tumor. It affects mostly young persons and has poor outcome with the present treatment. No improvement was observed since the introduction of chemotherapy. The better understanding of osteosarcoma development could indicate better management strategy. Repetitive DNA elements were found to play a role in cancer mechanism especially in epithelial tumors but not yet analyzed in osteosarcoma. We conducted the study to analyse the expression profile of repetitive elements (RE) in osteosarcoma.

**Methods:** Fresh bone paired (tumor and normal bone) samples were obtained from excised parts of tumors of 18 patients with osteosarcoma. We performed sequencing of RNA extracted from 36 samples (18 tumor tissues and 18 normal bone for controls), mapped raw reads to the human genome and identified the REs. EdgeR package was used to analyse the difference in expression of REs between osteosarcoma and normal bone.

**Results:** 82 REs were found differentially expressed (FDR < 0.05) between osteosarcoma and normal bone. Out of all significantly changed REs, 35 were upregulated and 47 were downregulated. HERVs (*THE1C-int, LTR5, MER57F* and *MER87B*) and satellite elements (*HSATII, ALR-alpha*) were the most significantly differential expressed elements between osteosarcoma and normal tissues. These results suggest significant impact of REs in the osteosarcoma. The role of REs should be further studied to understand the mechanism they have in the genesis of osteosarcoma.

## Impact statement

Osteosarcoma is a very aggressive bone cancer. It is rare but it touches dominantly adolescence. No changes in outcome of the present treatment for this poor prognosis disease makes additional research necessary. We sequenced total RNA from 36 fresh-frozen paired samples (18 tumor and 18 non-tumor) from osteosarcoma patients. We found 82 repetitive DNA elements (REs) expressed differently between osteosarcoma and normal bone. We believe that the results would contribute to the understanding of OS and provide new approach for further studies in this area.

## Introduction

Osteosarcoma is a rare cancer which affects 0.2–0.35/100 000 across countries with a slightly higher frequency in male than female (Mirabello et al., 2009a; Valery et al., 2015). It is the most common malignant bone tumor (Mirabello et al., 2009b). The highest peaked incidence was observed in women at 10–14 years and in men at age 15–19 years worldwide. There is a lower second peak in elderly (Mirabello et al., 2009a; Hung et al., 2014). The combination of surgery and chemotherapy remains standard of care. Introduction of effective chemotherapy during 1960s−1980s increased the cure rate from < 10% to 60–75% but no more gain later on (Jaffe et al., 2013). Many studies were carried out for understanding more about the mechanism of osteosarcoma and to look for more new effective treatment.

The initial sequencing of the human genome in 2001 showed that repetitive DNA sequences accounted for at least 50% of the genome (Lander et al., 2001). But more recent computational approaches stated a higher proportion as two-thirds of repetitive elements in the human genome (de Koning et al., 2011). They were classified into five classes: transposon-derived repeats, simple sequence repeats; segmental duplications; blocks of tandemly repeated sequences and ribosomal gene clusters (Lander et al., 2001). They can also be classified into two broad classes: tandem repeats and transposable elements (Padeken et al., 2015). Tandem repeats included satellite DNA, minisatellite and microsatellite (Padeken et al., 2015). While transposable elements were classified into retrotransposon (class I) and DNA transposons (class II). About 45% of genome belongs to transposon-derived repeats. Class I retrotransposons includes long terminal repeat (LTR)/human endogenous retroviruses (HERVs) and non-LTR retrotransposons such as LINEs (Long Interspersed Nuclear Elements) and SINEs (Short Interspersed Nuclear Elements) (Lander et al., 2001; Rebollo et al., 2012). Human endogenous retroviruses (HERVs) are a family of viruses integrated in our genome which have similarities with the nowadays exogenous retroviruses (Nelson et al., 2003). HERVs account for about 8% of human DNA (Lander et al., 2001; Cegolon et al., 2013). They are typically composed of gag, pol and env regions sandwiched between the two LTRs (long terminal repeats) (Nelson et al., 2003; Bannert and Kurth, 2004; Mager and Stoye, 2015).

Repetitive elements are found to be associated to some epithelial cancers. Overexpression of CT (centromeric) and PCT (pericentric) sequences was found in cancer tissues compared to normal tissues of the same patient who got testicular, liver, ovarian, and lung cancers (Eymery et al., 2009). Digital gene expression analysis of 15 pancreatic ductal carcinomas showed a median 21-fold increased expression of total amount of all satellite transcripts compared with normal pancreas. And the pericentromeric human satellite II (*HSATII*) was the biggest differentially expressed satellite subfamily which was not able to be detected in normal human pancreas and was expressed minimally in other normal tissues. Overexpression of *HSATII* was also observed in other human cancers including lung (2 of 2), kidney (2 of 2), ovarian (2 of 2), and prostate (3 of 3) (Ting et al., 2011; Bersani et al., 2015). The presence of a level of *GSATII, TAR1*, and/or *SST1* satellite transcripts below the reference level indicates that subject has a tumor (Schiavetti et al., 2002; Ting et al., 2012; Bersani et al., 2015). Alpha human satellite DNA was overexpressed (43 times) in pancreatic cancer compared to normal pancreas (Ting et al., 2011). Transcripts from *HERV-K HML-2* have been found to be associated with many cancers such as melanoma (Schiavetti et al., 2002) leukemia and lymphoma (Contreras-Galindo et al., 2008) as well as tumors of the breast (Pichon et al., 2006; Wang-Johanning et al., 2007) testis (Pichon et al., 2006) and ovary (Wang-Johanning et al., 2007). The *HERV-E* family has been found to be correlated with prostate, kidney, ovarian and uterine cancers (Wang-Johanning et al., 2003; Gimenez et al., 2010). *HERV-H* sequences was found to be overexpressed in colorectal carcinogenesis (Pérot et al., 2015).

For non-LTR retrotransposons, *LINE-1* was found to be overexpressed in tumor samples of pancreatic and prostate cancers (Contreras-Galindo et al., 2008; Criscione et al., 2014). *De novo* L1 insertions were found in colorectal cancer (Solyom et al., 2012). L1-mediated retrotransposition was suggested a potentially crucial source of mutations that can decrease the tumor suppression of somatic cells in hepatocellular carcinoma (Shukla et al., 2013). Few *SINE* subfamily were expressed differently in prostate cancer (Criscione et al., 2014).

Interesting findings about repetitive elements especially on epithelial cancers led to approval of patent for using some kinds of REs as biomarker in detection, prognosis and follow-up of several carcinomas. It raised the question about REs expression in sarcoma, a different kinds of malignant diseases. These repeated DNA sequences are not yet analyzed in case of osteosarcoma and it would be necessary to analyse their impact on the disease. Hence, we conducted this study with aim to reveal the differential expression of repeated DNA elements in osteosarcoma.

## Materials and methods

### Patients

This study was carried out in accordance with the recommendations of The Helsinki declaration. The protocols and informed consent forms used in the study were approved by the Ethics Review Committee on Biomedical Research of Hue University of medicine and pharmacy. All the participants or representative of patients signed the informed consents after being explained about the study.

Eighteen Vietnamese patients who had histologically confirmed osteosarcoma and who were indicated for surgery (limb sparing or amputation) were involved. The normal bone and cancerous bone were sampled from the removal part right after the operation. All of these samples were transported with dry ice and were stored at −80°C until RNA extraction.

### RNA extraction from bone tissue

Forty to fifty milligrams of bone sample was grinded with nitrogen by pestle and mortar into powder and pre-treated with trizol. We used RNeasy Fibrous Tissue Mini Kit (Qiagen, Valencia CA, USA) to extract total RNA from bone tissue according to the manufacturer's protocol. Extracted RNA was dissolved in RNase free water and stored at −80°C. Agilent 2100 Bioanalyzer and the RNA 6000 Nano Kit (Agilent Technologies Inc., CA, USA) were used to measure the quality of total RNA.

By applying Ovation RNA-Seq System V2 (NuGen, Emeryville, CA, USA), Fifty nanograms of total RNA was amplified. The collected cDNAs were pooled in equal amount and we used the pool to prepare the DNA fragment library by using SOLiD System chemistry (Life Technologies Corp, Carlsbad, CA, USA). SOLiD 5500W platform and DNA sequencing chemistry (Life Technologies Corp., Carlsbad, CA, USA) were applied for sequencing. By using Maxmapper algorithm implemented in the Lifescope software (Life Technologies, Ltd), Raw reads (75 bp) were color-space mapped to the human genome hg19 reference using. It was permitted to map multiple locations. The mapping confidence was more than 90 because the quality threshold was set to 10. Reads were filtered out if the score was < 10. Mapping quality was at an average of 30. The RepEnrich pipeline with RepeatMasker hg19 library as reference was used for obtaining read counts based on reads aligning to repeated element loci.

### Statistical analysis

For statistical analysis, edgeR (empirical analysis of DGE-digital gene expression in R) package for R was used (Robinson et al., 2010). EdgeR is a bioconductor package in R used to analyse the differential expression of digital gene expression data. It was developed for RNA-seq or other counts data. It provides methods to analyse the differential expression by using the negative binomial distribution and a shrinkage estimator for the distribution's variance. The Package performs sample comparison and it can adjust the *P*-value to overcome problems of multiple testing (Li et al., 2012; McCarthy et al., 2012; Anders et al., 2013). EdgeR package uses Benjamini-Hochberg procedure to control the false discovery rate (FDR) (Benjamini and Hochberg, 1995).

## Results

### General characteristics of osteosarcoma patients in the study

We collected samples after surgical removal of affected bone from 18 Vietnamese osteosarcoma patients. Each surgically removed sample contained paired tumor and normal part. Pathologist confirmed the diagnosis after histological analysis. Among 18 patients, there were 06 (33.33%) females and 12 (66.67%) males. The mean age was 18.11, which ranged from 7 to 52 years. Affected sites were at femur 55.56%, tibia (33.33%) and humerus (11.11%). The Table 1 shows an overview of the involved OS patients.

**Table 1:** Characteristics of osteosarcoma patients in the present study.

**Patient code**	**Age at diagnosis**	**Gender**	**Site of tumor**	**Metastasis at diagnosis**	**Chemotherapy**
OSVN001	16	Female	Femur	No	Yes
OSVN003	13	Male	Femur	No	Yes
OSVN004	16	Female	Femur	No	Yes
OSVN005	18	Male	Femur	No	Yes
OSVN006	18	Male	Femur	No	Yes
OSHN008	24	Female	Tibia	No	yes
OSVN008	52	Male	Femur	Yes	No
OSHN009	16	Male	Femur	No	Yes
OSHN010	20	Female	Femur	No	Yes
OSHN011	07	Male	Tibia	No	Yes
OSHN012	11	Male	Humerus	No	No
OSHN013	17	Male	Femur	No	No
OSHN014	16	Female	Tibia	No	Yes
OSVN015	15	Male	Tibia	No	Yes
OSHN015	8	Female	Tibia	No	Yes
OSHN016	20	Male	Femur	No	Yes
OSHN017	16	Male	Humerus	No	Yes
OSDN001	23	Male	Tibia	Yes	Yes

### Repetitive elements/repeated sequence (repeats) were found differently expressed between osteosarcoma and normal bone tissue

We analyzed 1116 different repeated elements from the Repbase. Benjamini-Hochberg (BH) adjustment which is implemented in edgeR was used to eliminate the false positives. These values, called the BH-adjusted *p*-values (FDR), were demonstrated in the column FDR. Hence, if we consider that 10 percent is a acceptable fraction for false positives, we can consider all repetitive elements with an adjusted *p*-value < 10% = 0.1 as significant ones. Here we accepted the level of 5% false positives by using FDR < 0.05. Consequently, 82 repetitive elements were found differentially expressed between the normal and osteosarcoma tissues. Of which, 35 elements were upregulated and 47 were downregulated.

Ranking by FDR, we have the list of elements with the lowest FDR known as the most significant elements differentially expressed in OS samples compared to normal ones. Ten elements with the lowest FDR were shown in the Table 2. The highest significant ones were *THE1C-int, LTR5, MER57F, MER87B* which are all belong to HERVs, a part of repetitive elements. We will discuss more about these elements in the discussion part. We also have two satellites *HSATII* and *ALR_Alpha* in the list.

**Table 2:** Ranking from the lowest FDR, ten most significant REs expressed differentially between osteosarcoma and normal control, FDR < 0.001.

**Class**	**Family**	**Element**	**logFC**	***p*-value**	**FDR**
LTR	ERVL-MaLR	*THE1C-int*	0.612785	9.83E-10	1.07E-06
LTR	ERVK	*LTR5*	0.606272	4.58E-08	2.49E-05
LTR	ERV1	*MER57F*	0.943969	1.09E-07	2.98E-05
LTR	ERV1	*MER87B*	0.748537	1.01E-07	2.98E-05
RNA	RNA	*7SK*	0.9486	3.17E-07	6.91E-05
LTR	ERV1	*MER34B-int*	0.72914	4.78E-07	8.68E-05
LTR	Gypsy	*MamGypLTR3*	−0.41918	7.13E-07	0.000111
Satellite	centr	*ALR_Alpha*	1.291039	1.08E-06	0.000147
DNA	DNA	*MER136*	1.412429	1.27E-06	0.000154
Satellite	Satellite	*HSATII*	1.878344	3.66E-06	0.000366

*REs with the lowest FDR were THE1C-int, LTR5, MER57F, MER87B. These elements belong to long terminal repeats (LTR) class and they are all upregulated in OS (logFC > 0.5). ALR_Alpha and HSATII were the most significant satellites that expressed differentially between OS and normal control samples*.

Ranking by logFC, we got a list of the most upregulated elements (Table 3) which have the highest logFC (1.12–2.05). The most upregulated elements were *SAR, HSATII, _CATTC_n, MER136, ALR_Alpha, _GAATG_n*. The most downregulated elements were mostly HERVs (Table 4).

**Table 3:** Satellites were found to be the most upregulated elements in OS.

**Class**	**Family**	**Element**	**logFC**	***p*-value**	**FDR**
Satellite	Satellite	*SAR*	2.052918	5.08E−05	0.003461
Satellite	Satellite	*HSATII*	1.878344	3.66E−06	0.000366
Satellite	Satellite	*_CATTC_n*	1.481369	3.7E−06	0.000366
DNA	DNA	*MER136*	1.412429	1.27E−06	0.000154
Satellite	centr	*ALR_Alpha*	1.291039	1.08E−06	0.000147
Satellite	Satellite	*_GAATG_n*	1.115843	0.000789	0.01829

*By ranking logFC, the highest ones compose of SAR, HSATII, and -CATTC-n with high significance (FDR < 0.01)*.

**Table 4:** Ranking by the lowest logFC, the table includes the most downregulated repetitive elements in osteosarcoma.

**Class**	**Family**	**Element**	**logFC**	***p*-value**	**FDR**
DNA	TcMar	*MamRep1161*	−0.99091	6.38*E*−05	0.003862
tRNA	tRNA	*tRNA-Ser-TCY*	−0.94009	0.001438	0.026562
LTR	ERV1	*HERV1_LTRe*	−0.68333	0.000166	0.007247
LTR	ERVL-MaLR	*MLT1E1-int*	−0.64431	0.00306	0.043679
LTR	ERV1	*HERV15-int*	−0.61309	0.000353	0.010703
LTR	ERV1	*MER51E*	−0.5277	0.000964	0.02144
LTR	ERV1	*MER83B*	−0.43948	0.000292	0.010112
LTR	ERVL	*LTR47B4*	−0.43666	7.71E−05	0.0042
LTR	Gypsy	*MamGypLTR3*	−0.41918	7.13E−07	0.000111
LTR	ERV1	*MER72B*	−0.37858	0.000402	0.011198
LTR	Gypsy	*LTR81*	−0.35087	0.003015	0.043679
LTR	ERV1	*LTR31*	−0.34699	0.000763	0.01808

*They are mostly HERVs*.

**Figure 1:**
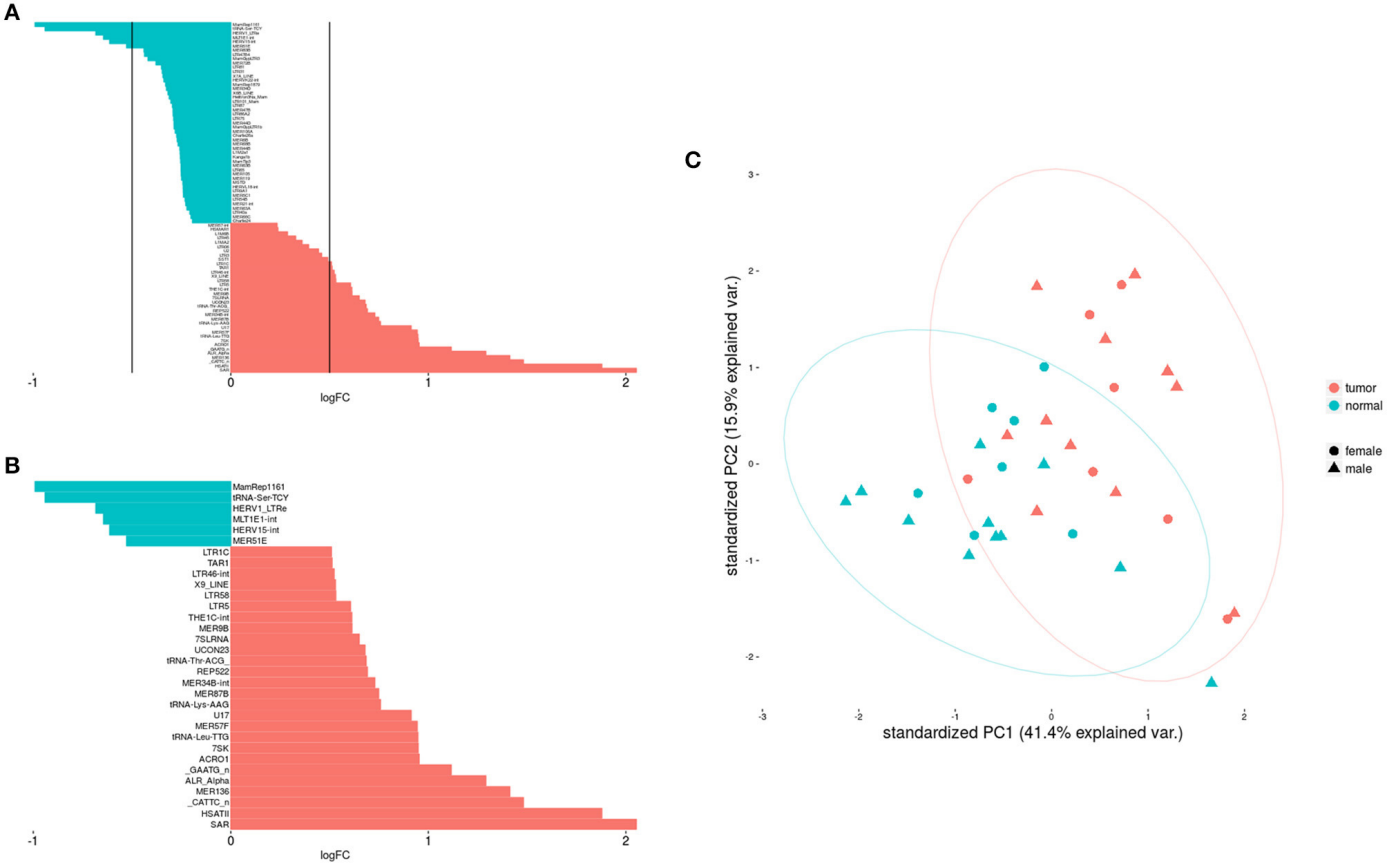
Differential expression of repetitive elements in OS was shown here. **(A)** Shows differentially expressed repetitive elements with significance while **(B)** Shows only elements expressed differentially with |logFC| > 0.5. **(C)** Shows the difference between tumor and non-tumor samples about REs expression.

**Figure 2:**
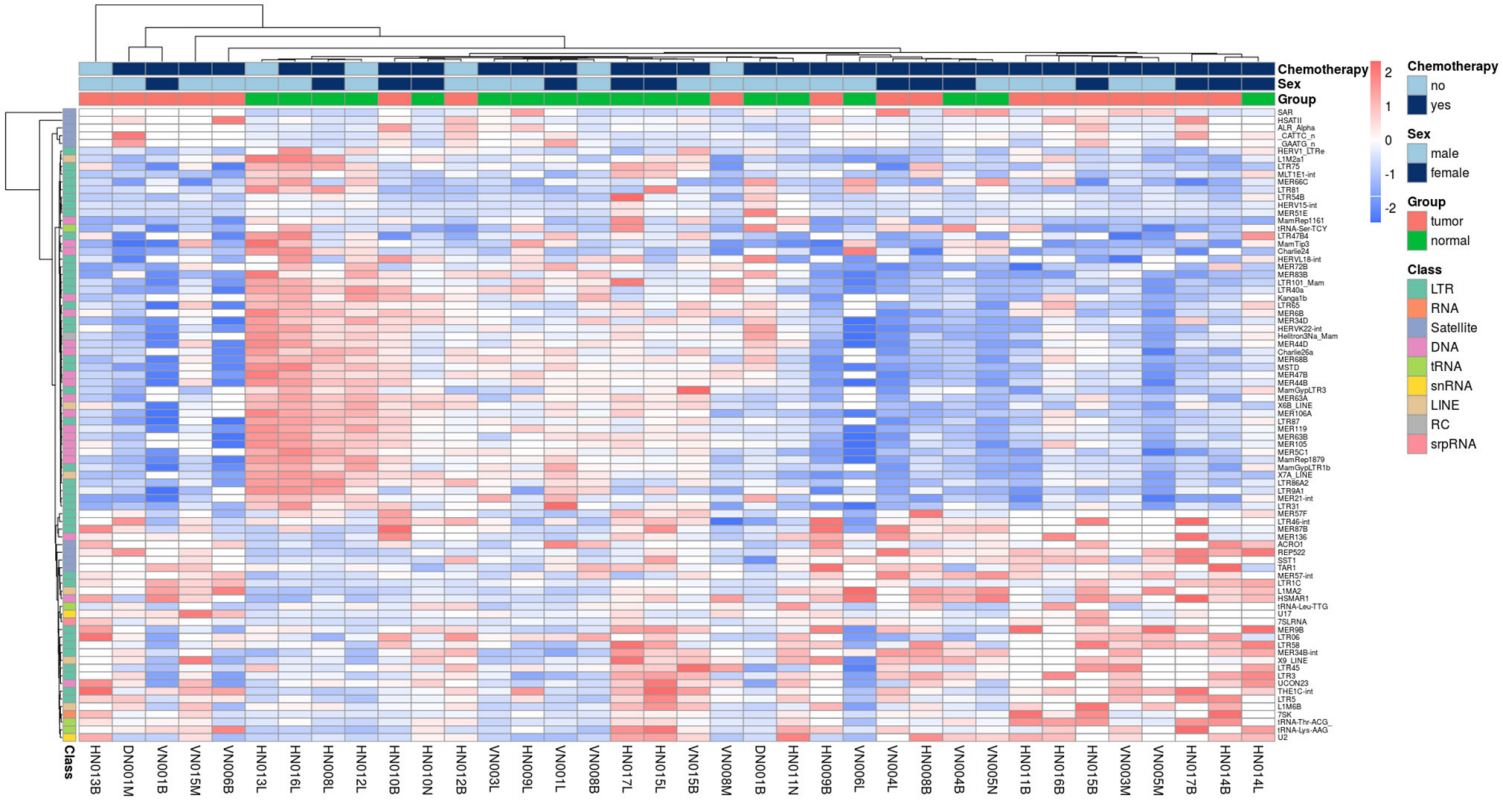
Heatmap of REs expression in 18 paired- fresh-bone samples stratified by tumor and non-tumor group, class of REs, gender, and chemotherapy vs. non-chemotherapy treated.

**Figure 3:**
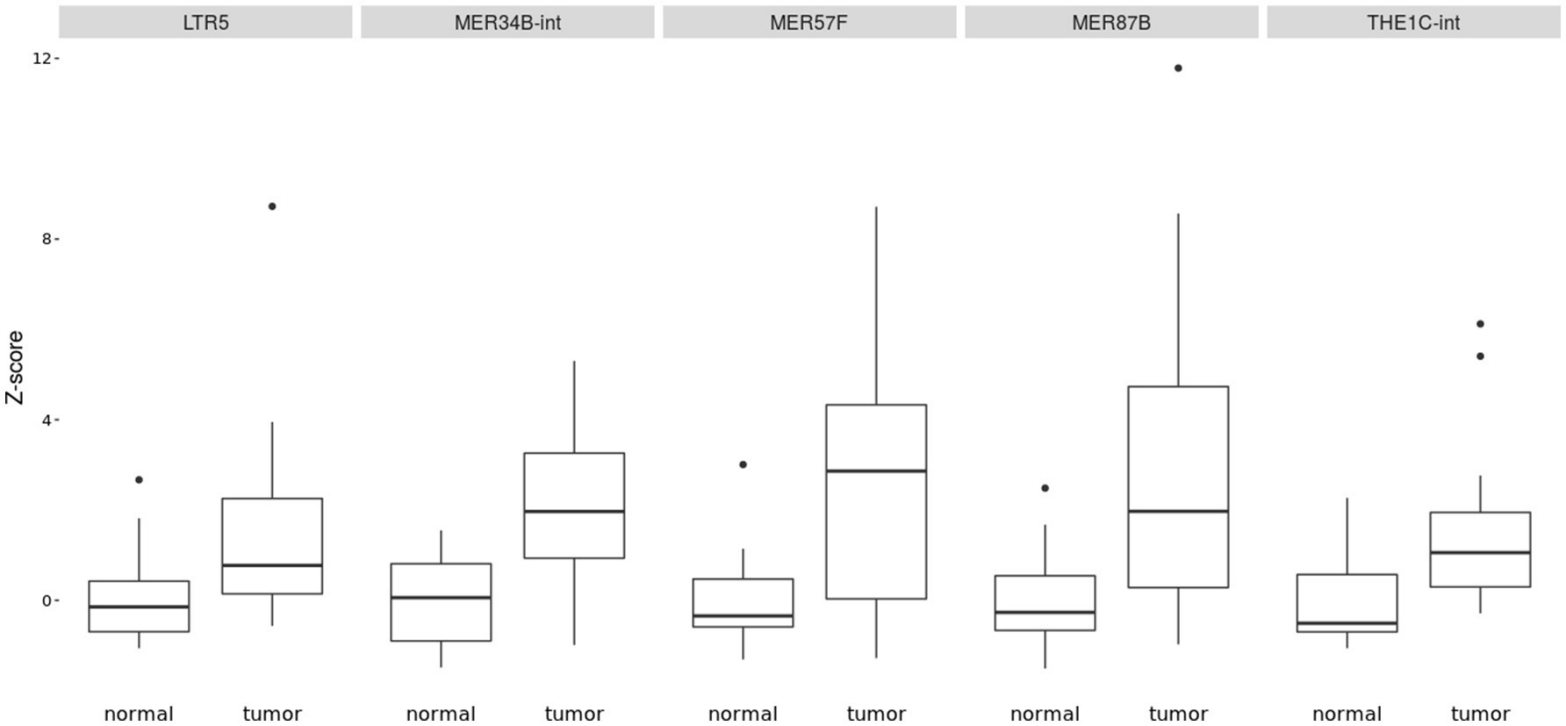
Boxplot of z-score comparing tumor vs. normal samples in some most significant REs expressed in OS (*LTR5, MER34B-int, MER57F, MER87B, and THE1C-int*). They were all upregulated in affected bone vs. normal bone.

The Figure 1 shows clearly the upregulated and downregulated repetitive elements in OS where we can see the most upregulated and the most downregulated ones with the range of logFC (Figures 1A,B). PCA shows nicely the difference between OS and normal bone in repetitive elements expression (Figure 1C). Figure 2 is a heatmap of differentially expressed repetitive elements at FDR ≤ 0.05 in OS vs. normal bone tissue. We can see clear pattern that differentiate between tumor and normal samples. Z-scores of the top five differentially expressed repetitive elements with lowest FDR values representes as boxplots in the Figure 3.

## Discussion

To our present knowledge, no similar study was carried out in osteosarcoma. We found herein 82 repetitive elements which were expressed differentially with significance FDR < 0.05 between osteosarcoma and adjacent normal bone in paired samples. Of which, 35 were upregulated and 47 were downregulated with FDR < 0.05.

The top most significant differentially expressed repetitive elements (with lowest FDR) were *THE1C-int, LTR5, MER57F, and MER87B*. Interestingly, they all belong to LTR elements or HERVs with DNA transposons made up of transposable elements (TEs). *THE1C-int* is a ERV3, a retrovirus-like MaLR element, consisting of 375 bp. *LTR5* is ERV2, clone of *HERV-K18* consisting of 969 bp. *MER57F* is a ERV1 with 435 bp and *MER87B* is also a ERV1 of 509 bp (Jurka et al., 2005; Bao et al., 2015). To our knowledge, *THE1C-int, MER57F, and MER87B* were not yet mentioned to be correlated with diseases in previous literature. While *LTR5* hypomethylation was found to be correlated to systemic lupus erythematosus mechanism (Nakkuntod et al., 2013). Many studies and observations suggested an important role of transposable elements in genomic instability, transcriptional control, non-coding RNA regulation, oncogenic activation and chromosomal rearrangements, Anwar et al. (2017). HERVs was mentioned a lot recently as a TE that may have a role in carcinogenesis in many different cancers that we got some evidences based on present literature.

In prostate cancer, *HERV-E* (and/or ERV3) env genes was expressed only in prostate tumor cells that suggested as targets for immunotherapy for the disease (Wang-Johanning et al., 2003). *HERV-K* was also identified to be involved to prostate cancer. It was found that forward and reverse transcripts of several *HERV-K* loci were detected in prostate cancer cell lines (Agoni et al., 2013) and overexpression of *HERV-K* (22q11.23) was associated with hypomethylation of the *HERV-K* locus in the disease (Goering et al., 2011). Interestingly, in a study of 14 patients with different grades of prostate cancer in paired samples (prostate tumor and normal tissue), 475 retrotransposon subfamilies were detected to be significantly differential expression in tumor tissue with FDR < 0.05. Among thoses elements, LTR was the most prevalent and most of them were endogenous retroviruses with *ERV1* being the most represented (Ren et al., 2012; Criscione et al., 2014).

In ovarian cancer, HERV-K env protein was expressed in epithelial ovarian cancer with high frequency (90%) without expression in normal and benign ovarian surface epithelial tissue. Interestingly, *ERV3* and *HERV-E* were found to expressed simultaneously in the same ovarian cancer tissues and antibodies to HERVs were present in the sera of ovarian cancer. Authors suggested that HERVs should be further studied to provide a new ovarian cancer screening tool and potentially serve as a new target for detection, diagnosis and treatment for this cancer (Wang-Johanning et al., 2007). Methylation levels of HERV-K and *HERV-E* were decreased in advanced ovarian cancer. *HERV-K* hypomethylation was found to be correlated to ovarian cancer aggressiveness and poor response to treatment (Iramaneerat et al., 2011).

In breast cancer, HERV-K env was suggested to be a tumor marker as it was expressed only in breast cancer tissues and cell lines analyzed but not detected in normal breast tissues (Wang-Johanning et al., 2001). HERV-K Env proteins were expressed in more than 85% cases of breast cancer patients which induced both serologic and cell-mediated immune responses (Wang-Johanning et al., 2008). High titers of of env RNA of *HERV-K (HML-2)* was found in the plasma of patients with breast cancer (Contreras-Galindo et al., 2008).

Transposable elements were also studied in hepato-gastrointestinal cancers. The *HERV-H* sequence was found to be overexpressed in up to 30–50% of different gastrointestinal cancers such as colorectal, gastric and pancreas cancers (Wentzensen et al., 2007). Additionally, the gag gene of a *HERV-H* locus on the chromosome Xp22 was commonly expressed in colon malignant tissues (Alves et al., 2008). LTRs were found to be upregulated and *LTR-007* was noted to be the most frequently expressed LTR in hepatocellular carcinoma (Hashimoto et al., 2015).

Additionally, RNA from human endogenous retrovirus K (*HERV-K) (HML-2)* can be found at a very high titers in the plasma of lymphomas patients and it decreased dramatically with succesful treatment of lymphoma (Contreras-Galindo et al., 2008). A very interesting study on tumorigenesis of Hodgkin's lymphoma provided evidence that endogenous LTR activation can be oncogenic and hypomethylation of the *THE1B LTR* (a MaLR family LTR retrotransposon, the same family THE1 with *THE1C-int* found in our study) caused the CSF1R (colony stimulating factor 1 receptor gene) oncogene activation. It was emphasized that CSF1R was strongly expressed in these malignant cells (Lamprecht et al., 2010).

HERVs were also studied in melanoma. HERV-K mRNA and proteins were found in melanoma tissues and cell lines (Muster et al., 2003; Büscher et al., 2005) and *HERV-K (HML-2)* loci being transcribed in melanoma tissues was identified (Schmitt et al., 2013).

HERVs was found in some germinal cancers. Production of *HERV-K* virus-like particle was identified in teratocarcinoma cell lines (Boller et al., 1993; Löwer et al., 1993); *HERV-K* Gag and Env proteins were expressed in germ cell tumors and interestingly antibodies against these proteins were also detected (Sauter et al., 1995, 1996). Hypomethylation of several HERV-W loci seems to be important in the *HERV-W* activation in testicular cancer (Gimenez et al., 2010).

Though many studies revealed the role of HERVs in oncogenesis, further studies need to be carried out for further understanding the direct role of these elements in cancer innitialization. Our findings here maybe the first in osteosarcoma and they match with the present understanding about transposable elements in cancer generally.

Ranking by logFC, we can generate some of the most upregulated repetitive elements with highest logFC (Table 3). Among those, *SAR, HSATII and -CATTC-n* were the top 3 most upregulated ones.

*SAR* is a human satellite I DNA consisting of 84 BP with sequence: acagtatata atatatattt tgggtacttt gatattttat gtacagtata taatatatat tttgggtact ttgatatttt atgt (Jurka et al., 2005; Bao et al., 2015). It has not been mentioned in previous studies on cancer. This finding may suggest for further looking for its role in osteosarcoma and we can discuss the function of *SAR* in the light of another satellite element *HSTAII*, that is pericentromeric human satellite II element. *HSTAII* was the second most upregulated element in osteosarcoma after SAR. *HSTAII* was found to be upregulated in some kinds of epithelial carcinomas such as pancreatic adenocarcinoma, lung cancer, renal cancer, ovarian, prostate cancer, colon cancer (Ting et al., 2011; Bersani et al., 2015). In another study analyzing 15 human pancreatic adenocarcinoma samples, 21-fold increased expression of all satellites transcripts compared with normal pancreas has been described. The highest differential expression was found for pericentromeric satellite *HSATII*. It was increased 131-fold in pancreatic cancer and undetectable in normal pancreas or very low expression in other normal tissues (Ting et al., 2011). Overexpression of *HSATII* was also found in other cancers such as lung cancer, kidney, ovarian and prostate. More interestingly, using RNA-ISH analysis of endoscopic ultrasound-guided fine-needle aspirates (EUS-FNA) of pancreatic masses revealed that HSATII-positive cells were detected in 10/10 cases confirmed adenocarcinoma in which 2 cases were non diagnostic with FNA (Ting et al., 2011). This is very promising for further study in the future on osteosarcoma with the present findings in epithelial carcinomas. *HSATII* copy gain in colon cancer was associated with a significant reduction in overall survival compared with no gain tumors (Bersani et al., 2015). Interesting experiment with colon cancer cells line treated by inhibiting *HSATII* rdDNA formation in 3D conditions and mouse tumor xenografts lead to tumor shrinking with reduction of *HSATII* copy number gain (Bersani et al., 2015).

Simple satellite repeat *(CATTC)n* was found to be overexpressed in human colon cancer simultaneously with *HSATII* and *ALR/alpha*. This finding completely supports our results where we found similar simultaneous upregulation of the REs (Bersani et al., 2015).

Taken together, our findings of changed expression of repetitive elements in osteosarcoma match with previous results in epithelial, germinal, and hematologic cancers. It suggests that REs could have potential biomarkers in detection, diagnosis and follow-up osteosarcoma. It needs further studies to verify this preliminary results.

We admitted that we have some limitations to be improved. Our samples were heterogenous but it was acceptable to make comparision between malignant group and control group due to paired samples. Moreover, in the Figure 2, we can see that patients without chemotherapy seem not to have higher similarity in their subgroup. But it is difficult to make a conclusion because only 3 patients did not receive chemotherapy in 18 patients totally. Anyway, it raised a question to be verified if the repetitive elements expression is changed with chemotherapy.

## Conclusion

Eighty two repetitive elements were found differentially expressed between the normal and osteosarcoma tissues. Of which, 35 elements were upregulated and 47 were downregulated. The most significant repetitive elements differentially expressed between OS and normal adjacent normal bone were HERVs (*THE1C-int, LTR5, MER57F*, and *MER87B*). *SAR, HSATII*, and simple repeat *(CATTC)n* were the most upregulated in OS. The result of this study complements the known findings about HERVs and satellite DNA expression in epithelial, germinal and hematologic cancers and it indicates an interesting point to be further studied in OS.

## Data availability

Our data are available at the GEO repository under accession number GSE99671.

## Author contributions

XH contributed in study designing, samples and clinical data collection, laboratory works, data analysis, and writing manuscript. VN contributed in samples collection, data registry, follow up patients and manuscript writing. PP, VL, HN, NL and LT contributed in samples collection, data registry and manuscript review. ER, EP, GK, and FL contributed in RNA extraction, sequencing, data analysis and manuscript writing. AM, KM, and SK contributed in study design, supervising, data analysis and manuscript writing.

### Conflict of interest statement

The authors declare that the research was conducted in the absence of any commercial or financial relationships that could be construed as a potential conflict of interest.
